# 

**Published:** 1824-03

**Authors:** 


### MONTHLY CATALOGUE OF MEDICAL BOOKS.

*** *It having been hinted to us that gentlemen, sending copies of their Works, prefer having their titles given at length, it is our intention, in future, to comply with this suggestion: but it is to be observed, that no books can be entered on this List except those sent to us for the purpose;—finding, in the lists hitherto transmitted, that the names of works have frequently been given as published, which have not appeared for weeks, or even months after.*

Essay on the Effects of Iodine on the Human Constitution; with Practical Observations on its Use in the Cure of Bronchocele, Scrofula, and the Tuberculous Diseases of the Chest and Abdomen. By W. Gairdner, M.D.—pp. 64. London, 1824.

The Animal Kingdom, arranged in Conformity with its Organization, by the Baron Cuvier, &c. With additional Descriptions of all the Species hitherto named, of many not before noticed, and other original Matter; by Edward Griffith, F.L.S. and others.—pp. 203; Plates. London, 1824.

Observations on Strictures of the Rectum and Colon, and other Affections which diminish the Capacity of the Intestinal Canal in those Parts; including Spasmodic Constriction of the Sphincter Ani, (with a Translation of Mons. Boyer's valuable Paper on that Complaint,) Hæmorrhoidal Tumors, (called Piles,) Excrescences, and Prolapsus Ani; accompanied with the Mode of Treatment, several Cases, and Engravings. By W. White, Member of the Royal College of Surgeons, London; Corresponding Member of the London Medical Society; and one of the Surgeons to the City Infirmary and Dispensary, Bath. Fourth Edition, much enlarged.—pp. 217. Bath, 1824.

Pathological Observations, Part I. On Dropsy, Purpura, and the Influenza of the latter end of the Year 1822, and beginning of that of 1823; and particularly on the Morbid Changes of the Blood, and their Influence on the Production and Course of these Diseases, illustrated by several Cases and Dissections. By Wm. Stoker, M.D. Edin. Licentiate of the King and Queen's College of Physicians in Ireland, senior Physician to the Fever Hospital and House of Recovery in Cork-street, &c. &c. &c.—pp. 244. Dublin, 1823.

The Opinions of Reviewers and of other learned Men, concerning the Works of Dr. William Balfour, Physician in Edinburgh.—pp. 12. Edinburgh, 1821.

---

N.B.—Mr. Lizar's Plates sent to us scarcely admit of review: they are well executed, the descriptive letter-press is accurate, and the whole work promises to be a useful addition to our Anatomical Systems.

### NOTICE TO CORRESPONDENTS.

Dr. BALFOUR's *Paper came too late for insertion in the present Number. We shall be glad to hear from him again.*

*All the other Communications have already been privately acknowledged.*

*We beg to inform* Dr. RENWICK, *that the words in question were entirely omitted in the MS. sent to us: we supplied what appeared wanting to complete the sense.*

